# Spatial patterns in the size of Chinese lizards are driven by multiple factors

**DOI:** 10.1002/ece3.7784

**Published:** 2021-06-27

**Authors:** Tao Liang, Zi Zhang, Wen‐ya Dai, Lei Shi, Chang‐hu Lu

**Affiliations:** ^1^ Wildlife conservation and utilization Nanjing Forestry University Nanjing China; ^2^ College of Animal Science Xinjiang Agricultural University Urumqi China

**Keywords:** Bergmann's rule, climate, ectotherm, gradient, mass, spatial pattern

## Abstract

**Background:**

For almost two centuries, ecologists have examined geographical patterns in the evolution of body size and the associated determinants. During that time, one of the most common patterns to have emerged is the increase in body size with increasing latitude (referred to as Bergmann's rule). Typically, this pattern is explained in terms of an evolutionary response that serves to minimize heat loss in colder climates, mostly in endotherms. In contrast, however, this rule rarely explains geographical patterns in the evolution of body size among ectotherms (e.g., reptiles).

**Location:**

China.

**Aim:**

In this study, we assembled a dataset comprising the maximum sizes of 211 lizard species in China and examined the geographical patterns in body size evolution and its determinants. Specifically, we assessed the relationship between body size and climate among all lizard species and within four major groups at both assemblage and interspecific levels.

**Results:**

Although we found that the body size of Chinese lizards was larger in warmer regions, we established that at the assemblage level, size was correlated with multiple climatic factors, and that body size–climate correlations differed within the four major groups. Phylogenetic analysis at the species level revealed that no single climatic factor was associated with body size, with the exception of agamids, for which size was found to be positively correlated with temperature.

**Main conclusions:**

Geographical patterns in Chinese lizard body size are driven by multiple factors, and overall patterns are probably influenced by those of the major groups. We suggest that our analyses at two different levels may have contributed to the inconsistent results obtained in this study. Further studies investigating the effects of altitude and ecological factors are needed to gain a more comprehensive understanding of the evolution of ectotherm body size.

## Graphical abstract

We present the most comprehensive database of body size among Chinese lizards. Spatial patterns in the size of Chinese lizards are driven by multiple factors.

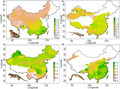

## 1 INTRODUCTION

In animals, body size is one of the most important life‐history traits, and geographical patterns in size distribution and its determinants are among the most intriguing features of macroecology (Pincheira‐Donoso & Meiri, 2013; Pincheira‐Donoso et al., 2019). In this regard, the influence of climate on the body size of endotherm and ectotherm groups has been well studied (Meiri & Dayan, 2003; Slavenko et al., 2019), given that climate has traditionally been considered a major driver promoting body size evolution (Olalla‐Tárraga and Rodríguez, 2007). Several hypotheses have been proposed to explain observed geographical patterns in the size distribution of ectotherms.

1. The “heat balance hypothesis” predicts that body size is more likely to increase with an increase in latitude, a characteristic referred to as Bergmann's rule, given that larger animals can produce more heat and lose relatively less heat (Bergmann, 1847; Freckleton et al., 2003; Meiri & Dayan, 2003; Olson et al., 2009). However, although this pattern is prevalent among endotherms, it rarely applies to ectothermic species such as small‐sized lizards (Olalla‐Tárraga, 2011; Penniket & Cree, 2015; Yu et al., 2019), with evidence for a reverse trend (Ashton & Feldman, 2003) or no trend (Pincheira‐Donoso & Meiri, 2013) often being reported for ectotherms. Accordingly, an alternative view predicts that body size decreases with a reduction in temperature, as rates of heating increase with reductions in the body size of large‐sized ectothermic species, such as snakes (Olalla‐Tárraga, 2011; Olalla‐Tárraga et al., 2006).
2. The “seasonality hypothesis” (see Slavenko et al., 2019 for “starvation resistance hypothesis”) predicts that body size increases with an increase of seasonality, given that accumulation of nutritional reserves at a larger size can enhance tolerance to changing climates (Boyce, 1979; Lindsey, 1966). However, this assumption does not take into consideration the short growing periods typical in certain seasonal zones, which may not be conducive to the development of a large size (Horváthová et al., 2013; Slavenko et al., 2019). Accordingly, body size could also decrease in response to increasing seasonality owing a reduction in the period of activity.
3. The “net primary productivity hypothesis” (NPP, also referred to as the “resource rule”) predicts that the availability of food resources (Rosenzweig, 1968) could be associated with higher productivity, thereby enabling the development of larger body size(Huston & Wolverton, 2011; Meiri et al., 2010; Yom‐Tov & Geffen, 2006).
4. The “water availability hypothesis” (Ashton, 2002) predicts that in arid areas, bodies of a larger size can preserve water more efficiently, owing to smaller surface‐area‐to‐volume ratios, as has been reported in amphibians (Pincheira‐Donoso et al., 2019).

Reptiles provide good models for research on geographical patterns in body size–climate correlations. With the exception of Antarctica, reptiles are extensively distributed on all continents over a diverse range of climatic zones (Feldman & Meiri, 2014), and their body temperature is largely dependent on environmental conditions (Lillywhite, 1987). Accordingly, climate may play a key role in shaping the life‐history traits of reptiles at the macro level. Indeed, a number of studies have revealed that the development of different ectotherm lineages could be related to different climatic factors, owing to differences in geographical (see global study of reptiles, Slavenko et al., 2019) and ecological (e.g., snakes, Feldman & Meiri, 2014) factors. Nevertheless, the findings of a recent study, conducted at the global level, tend to indicate that climatic factors alone cannot be used to predict lizard size (Slavenko et al., 2019). Moreover, lumping all species together at a large scale has proved to be inappropriate for examining the relationships between size and climate (Feldman & Meiri, 2014), given that size–climatic factor relationships might differ among lineages because of different ecological traits (but see Guo, 2016 for Chinese lizards). Climates can, however, be a stronger predictor of lizard size at finer scales (but see anoles, Velasco et al., 2020); for example, at the family scale, the body size of Tropidurinae lizards has been shown to be driven by precipitation (Brandt & Navas, 2013). Thus, it is necessary to evaluate whether patterns occur consistently at finer scales.

Although to date the patterns of reptile body size–climate correlations have been studied in North America (Olalla‐Tárraga et al., 2006; Tarr et al., 2019), Europe (Olalla‐Tárraga et al., 2006), and Australia (Feldman & Meiri, 2014), the geographical variation in lizard body size at the macro level has rarely been examined in China (but see Guo, 2016 for lizards at inter‐ and intraspecific level). This represents a significant omission, given that China is considered an ecologically important evolutionary domain for studying the pattern of body size–climate correlations of lizards because of the country's wide climatic trends across both latitudinal (3.85°–53.56°) and altitudinal (from −154.31 m to 8,848.86 m) gradients. These provide diverse climate zones for lizards to inhabit (but lizards extremely record for altitude is ~6,000 m, e.g., for *Phrynocephalus erythrurus*, Zhao et al., 1999). Moreover, China is populated by a large number of lizard species, which are diverse in their phylogenetic relationships (212 species belong to 10 families, Wang et al., 2020; Zhao et al., 1999; Zhou et al., 2019).

In this study, we sought to investigate the relationships between body size and climate among Chinese lizards. Specifically, we aimed to (1) examine the spatial patterns in body sizes among these lizards, (2) test the aforementioned four hypotheses proposed to account for relationships between body size and climate, and (3) determine whether there is consistent support for these hypotheses at the phylogenetic and familial scales.

## 2 MATERIAL AND METHODS

### 2.1 Data

**FIGURE 1:**
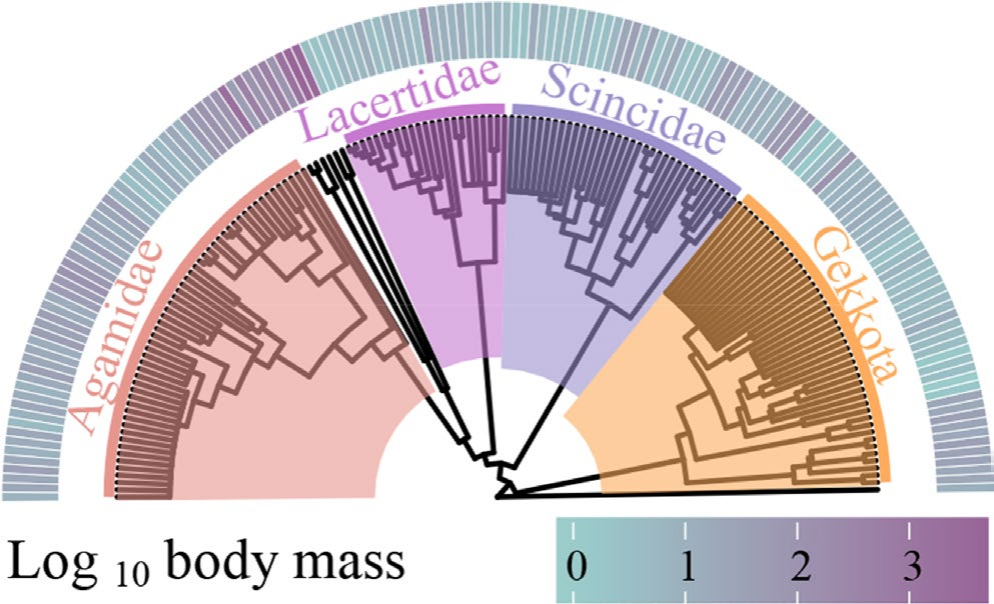
Phylogenetic relationship of body mass for 164 lizard species of China. Graph was created using the packages “ggtree” (Yu et al., 2017)

For the purposes of this study, we assembled the largest dataset of Chinese lizards (211 of 212 species) for body size that has been reported to date (body size data available from Dryad, datadryad.org, https://doi.org/10.5061/dryad.j6q573ndn.). In this regard, we followed the taxonomy of Wang et al. (2020) and Uetz et al. (2019). The lizards belong to 10 families, which can be assigned to four major family‐based groups: Agamidae (*n* = 67), Lacertidae (*n* = 31), Scincidae (*n* = 41), and Gekkota (Eublepharidae, *n* = 12; Gekkonidae, *n* = 48; Sphaerodactylidae, *n* = 3). Species in the families Anguidae (*n* = 3), Dibamidae (*n* = 2), Shinisauridae (*n* = 1), and Varanidae (*n* = 3) were excluded from the major groups, owing to small sample sizes. Phylogenetic data were obtained from the latest published global squamate maximum likelihood phylogenic tree (Tonini et al., 2016). Having removed species for which there were no morphological data, the remaining 164 out of 211 species were used for the following phylogenetic‐informed analysis (Figure 1). In this study, we obtained maps of the distribution for all 211 species, 165 of which were obtained from Roll et al. (2017), whereas those for the remaining 46 species were produced as part of the present study (see Appendix S1 for details).

The largest recorded snout–vent length (SVL) was used as the proxy measurement of body size, as it provides a good representation of the potential sizes attainable by squamates (Slavenko et al., 2019), and this is also the most commonly reported metric for Chinese lizards. Data were collected from the published literature, both research articles and books, and the SVL of some specimens obtained from Xinjiang Agricultural University (maximal data of seven species). We used “transformed‐mass” as a measure of body size presented in terms of clade‐specific length–mass allometric equations, which were obtained from the studies of Feldman et al. (2016) and Meiri (2010). This is because (1) SVL size cannot be compared directly between clades that differ markedly with respect to body plan (e.g., lizards, Feldman et al. 2016), and (2) Bergmann's rule proposed to explain the environmental clines in body size is based on body mass, not length (Blackburn et al. 1999; Santini et al. 2018).

We used five climate variables to investigate the role of environmental factors as drivers of geographical patterns in lizard size: (1) mean annual temperature; (2) temperature seasonality; (3) annual precipitation; (4) precipitation seasonality (PS); and (5) net primary productivity (NPP). The first four variables were from high‐resolution climatologies (years from 1901 to 2016) for the Earth's land surface areas (CHELSA, http://chelsa‐climate.org/), whereas NPP data were obtained from the Moderate Resolution Imaging Spectroradiometer database (MODIS, http://www.ntsg.umt.edu/project/mod17). We evaluated the veracity of the four hypotheses using two approaches (assemblage‐ and species‐level) with R software (version 3.6.1; R core team, 2019), based on analyses of all lizards (211 species) and within the aforementioned four taxonomic groups (see below).

### 2.2 Data analysis

#### 2.2.1 Assemblage‐based analysis

We used an assemblage‐based approach (Olalla‐Tárraga et al., 2010) by overlaying a grid of 1 × 1 (latitude–longitude) resolution as observational units (R package: LetsR, see Vilela & Villalobos, 2015). We rasterized the median body mass of species overlapping each grid cell (Meiri & Thomas, 2007) and computed the mean climate variables within each cell. Modeling was performed using spatial autoregressive (SAR) models (see review by Dormann et al., 2007) to account for spatial autocorrelation, with median body mass as the response variable, and the five environmental variables as predictors (*errorsarlm* function in “spdep” package, Bivand & Wong, 2018). This was performed for all 211 lizard species. We also performed modeling separately for each of the four major family‐based groups to determine whether the pattern of body size–climate correlations was consistent at a finer scale (within each of the four major groups).

We also took into account the fact that, at the assemblage level, repeated species co‐occurrences could generate unreliable relationships between size and climate (Hawkins et al., 2017) and thus indicate a departure from actual relationships. Consequently, we used a null modeling approach by randomizing the median body mass among species and generating 100 random spatial body size gradients. Modeling of these 100 randomized body size gradients was performed using the aforementioned SAR models, and we further evaluated the difference between the observed and 100 random Nagelkerke pseudo‐R^2^ value‐based single‐sample *t* tests. If the 100 random Nagelkerke pseudo‐R^2^ values were significantly lower than the observed values, we considered the observed pattern to be reliable (Hawkins et al., 2017).

#### 2.2.2 Species‐level analysis

To determine whether patterns of body size–climate correlations were consistent at the species level, we used multiple regression on phylogenetic generalized least square (PGLS), with log_‐10_‐transformed mass as the response variable, and the five environmental variables as the predictors (using *pgls* function in caper package, Orme et al., 2012). Analyses at the species level can also avoid errors attributable to repeated species co‐occurrences.

PGLS was performed for all assessed lizards (164 species) and separately for the four major groups. The remaining 47 species were excluded from the PGLS model owing to a lack of phylogenetic data. Therefore, we repeated the aforementioned analysis using generalized linear mixed models (GLMMs) with species as a random variable nested within genera, which, in turn, were nested within families (including 211 species). We further reran the mixed model analyses after removing 10% of species with the largest distribution ranges (see reviewed in Slavenko et al., 2019) to account for spatial variation within wide‐ranging species.

## 3 RESULTS

**FIGURE 2:**
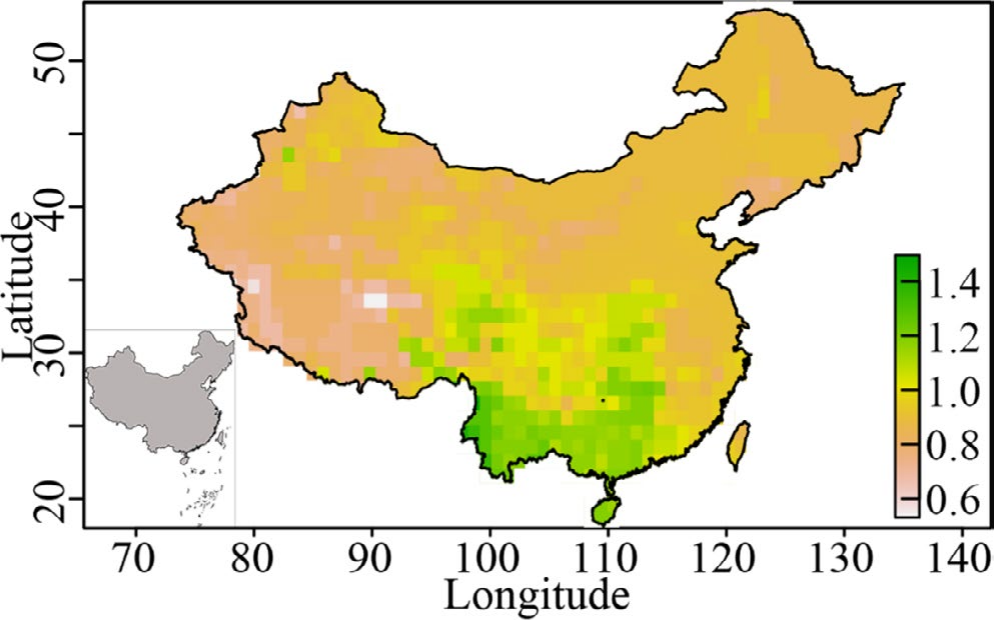
Geographical body size gradients for lizards across China. The body size of species in each grid cell was calculated as the median body size (log_10_ body mass) of all species occurring in that cell

We estimated that for 102 and 90 species, males and females have a higher maximum mass, respectively, whereas we were unable to determine a gender‐related difference for the remaining 19 species. Lizards with larger body sizes were found to be distributed mainly in regions in southern China (although not at high altitudes), whereas smaller sized lizards tended to be found in the north and at higher altitudes (Figure 2).

**FIGURE 3:**
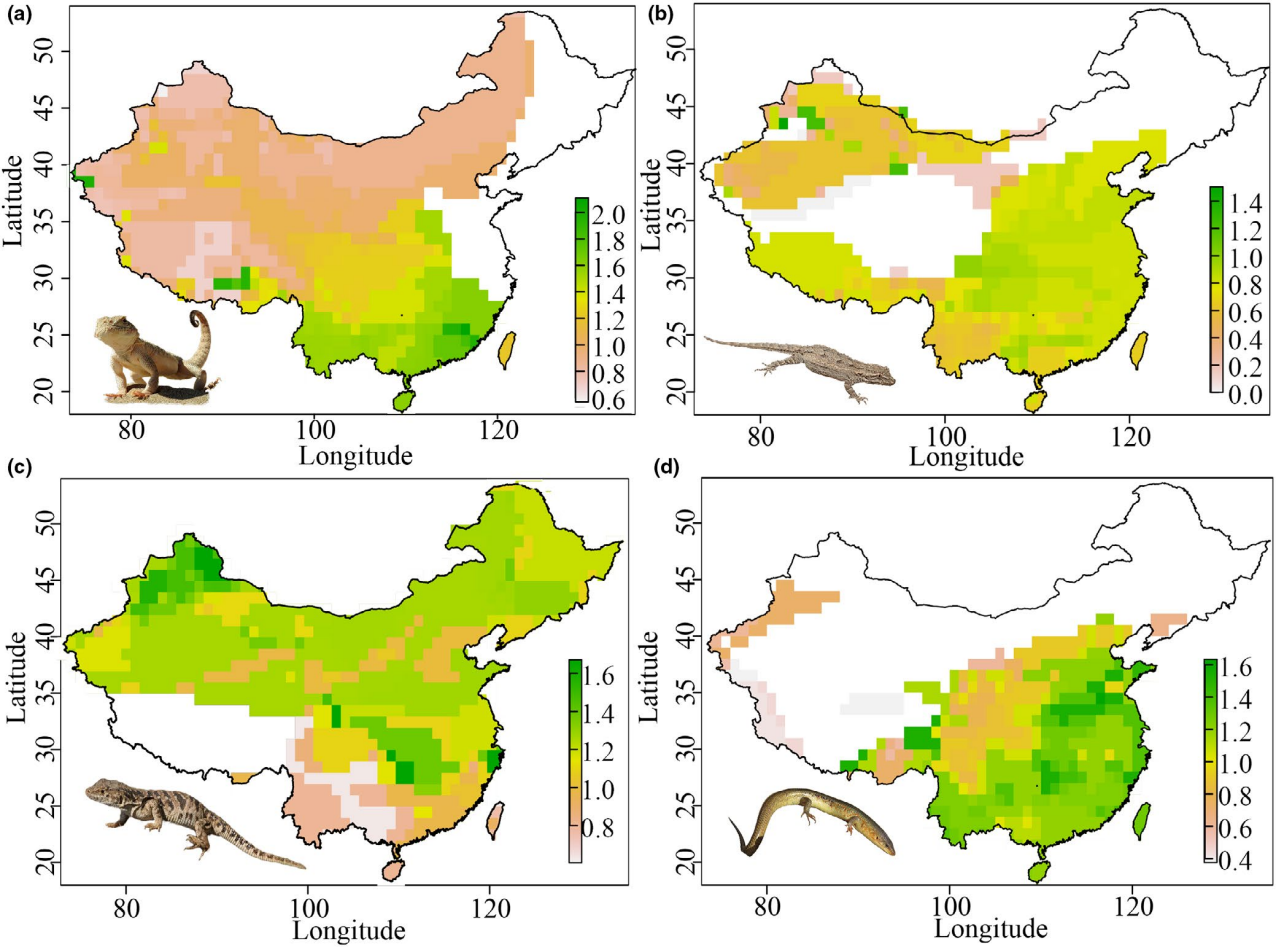
Geographical distribution of body size gradients among four major groups of lizards across China ((a) Agamidae; (b) Gekkota, (c) Lacertidae, and (d) Scincidae). The body size in each grid cell was calculated as the median body size (log_10_ body mass) of all species occurring in that cell. Four representative species were selected randomly within each group: A. *Phrynocephalus mystaceus*, (b) *Mediodactylus russowii*, (c) *Plestiodon chinensis,* and (d) *Eremias arguta*, respectively (Photographs: Tao Liang)

At the assemblage level, we detected an inverse pattern with respect to Bergmann's rule (i.e., a reduction in size with increasing latitude) in lizards as a whole. Most of the major groups also followed inverse Bergmann's rule, with only the distribution of lacertids conforming to Bergmann's rule (*p* < .05 in all cases; Table 1 and Figures 2 and 3), which states that size increases with increasing latitude at the species level. However, among the remaining three major groups and for lizards in general, we detected no significant correlations between size and latitude (Table 1).

**TABLE 1:** Latitudinal gradients of body size among Chinese lizards

Taxa	Estimate	*S.E*.	*R^2^*	*p*	lambda
Assemblage level					
Lizards (*n* = 211)^a^	−0.004	0.001	.61^c^	**<.001**	–
Agamidae (*n* = 67)	−0.024	0.002	.62^c^	**<.001**	–
Gekkota (*n* = 63)	−0.005	0.003	.17^c^	.06	–
Lacertidae (*n* = 31)	0.008	0.001	.49^c^	**<.001**	–
Scincidae (*n* = 41)	−0.019	0.004	.55^c^	.**049**	–
Species level					
Lizards (*n* = 164)^b^	−0.001	0.005	<.01	.842	0.98
Agamidae (*n* = 50)	−0.012	0.012	<.01	.324	0.917
Gekkota (*n* = 43)	−0.005	0.009	<.01	.602	1
Lacertidae (*n* = 26)	0.024	0.008	.23	.**012**	0.97
Scincidae (*n* = 33)	0.004	0.012	<.01	.717	1

Note

a,b: Nine and three species that were included in all lizards (*n* = 211, *n* = 155, respectively) were not included in the four major groups. c: Nagelkerke pseudo‐*R* ^2^, but these cannot be interpreted as the percentage of variance explained by the model. Significant relationships are in boldface.

**FIGURE 4:**
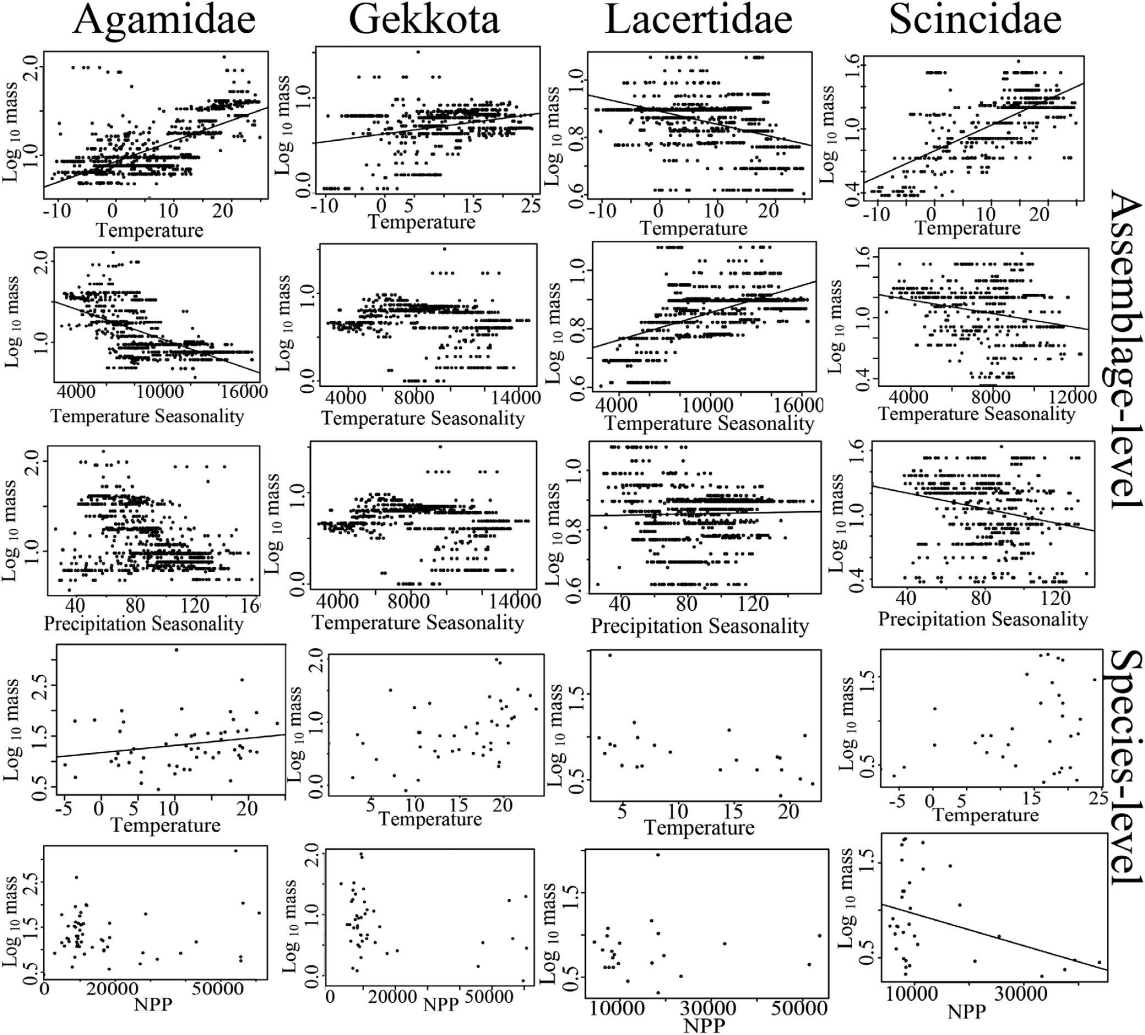
Multiple relationships between environmental variables and body size within major groups of Chinese lizards at assemblage and species levels. Black lines correspond to the fitted regression model (spatial autoregressive model for assemblage level and generalized linear mixed model for species level). Regression lines with *p* > .05 were not plotted

At the assemblage level, we found that body mass was positively correlated with precipitation and NPP in lizards as a whole. Whereas within the major groups, body size was correlated with temperature in all groups (negatively in lacertids, but positively in the remaining three groups); however, in none of the four groups was size correlated with precipitation. Furthermore, with the exception of geckos, either temperature or precipitation seasonality was correlated with body mass in other groups and NPP was correlated with mass only in agamids (Table 2; Figure 4). The null model approach performed here revealed that the observed Nagelkerke pseudo‐R^2^ values were significantly larger than the random values except for geckos, and consequently, we considered the detected gecko body size–climate correlations to be spurious (Appendix S2).

**TABLE 2:** Results of spatial autoregressive analyses at assemblage and species levels

	Temperature	Temperature seasonality	Precipitation seasonality	Precipitation	Net primary productivity	*R* ^2^
Assemblage level						
Lizards (*n* = 211)^a^	1.0e−3 ± 6.8e−4^n.s.^	−2.05e−7 ± 2.7e−6^n.s.^	−7.6e−5 ± 1.5e−4^n.s.^	**2.8e−5 ± 1.0e−5** ^**^	6.4e−6 ± 2.6e−6^*^	.62^c^
Agamidae (*n* = 67)	5.5e−3 ± 1.4e−3^***^	−1.78e−5 ± 5.8e−6^**^	−5.6e−4 ± 3.2e−4 ^n.s.^	6.4e−06 ± 2.1e−5 ^n.s.^	1.4e−05 ± 5.6e−6^*^	.71^c^
Gekkota (*n* = 63)	4.6e−3 ± 2.0e−3^*^	5.62e−6 ± 7.7e−6 ^n.s.^	−6.2e−5 ± 3.9e−4 ^n.s.^	−1.4e−6 ± 2.6e−5^n.s.^	−1.1e−7 ± 7.4e−6^n.s.^	.19^c^
Lacertidae (*n* = 31)	−2.4e−3 ± 5.6e−4^***^	**1.62e−5 ± 2.2e−6** ^***^	**−8.6e−4 ± 1.3e−4** ^***^	1.0e−5 ± 1.0e−5^n.s.^	3.6e−06 ± 2.3e−6^n.s.^	.52^c^
Scincidae (*n* = 41)	1.1e−2 ± 2.7e−3^***^	**−3.58e−5 ± 1.0e−5** ^***^	**−1.3e−3 ± 6.1e−4** ^*^	−4.2e−05 ± 2.8e−5^n.s.^	−1.4e−05 ± 7.8e−6^n.s.^	.56^c^
Species level: a						lambda
Lizards (*n* = 164)^b^	**0.017 ± 0.006** ^**^	8.04e−6 ± 1.84e−5^n.s.^	1.88e−3 ± 1.65e−3^n.s.^	−6.82e−5 ± 7.4e−5^n.s.^	−2.5e−6 ± 2.4e−6^n.s.^	.983
Agamidae (*n* = 55)	0.012 ± 0.008^n.s.^	**−8.3e−05 ± 3.3e−5** ^*^	−3.6e−3 ± 2.4e−3^n.s.^	−1.1e−4 ± 8.1e−5^n.s.^	−4.1e−7 ± 3.3e−6^n.s.^	.891
Gekkota (*n* = 45)	0.031 ± 0.016^n.s.^	6.3e−06 ± 5.0e−5^n.s.^	3.9e−4 ± 4.3e−3^n.s.^	−2.1e−4 ± 1.9e−4^n.s.^	−9.1e−6 ± 5.9e−6^n.s.^	1
Lacertidae (*n* = 26)	0.004 ± 0.021^n.s.^	2.7e−5 ± 3.2e−5^n.s.^	−5.6e−3 ± 2.6e−3^n.s.^	−2.1e−4 ± 1.4e−4^n.s.^	3.9e−6 ± 4.8e−6^n.s.^	1
Scincidae (*n* = 33)	0.029 ± 0.031^n.s.^	−2.9e−5 ± 5.3e−5^n.s.^	4.1e−3 ± 7.5e−3^n.s.^	−3.4e−4 ± 3.8e−4^n.s.^	−1.3e−5 ± 1.6e−5^n.s.^	.62
Species‐level: b^d^						
Lizards (*n* = 211)^a^	**0.011 ± 0.005** ^*^	−1.09e−6 ± 1.57e−5^n.s.^	7.2e−4 ± 1.47e−3^n.s.^	−7.04e−5 ± 6.02e−5^n.s.^	−1.3e−6 ± 2.02e−6^n.s.^	–
Agamidae (*n* = 67)	**0.018 ± 0.008** ^*^	−1.2e−6 ± 3.1e−05^n.s.^	2.6e−03 ± 2.2e−03^n.s.^	−8.8e−06 ± 8.9e−05^n.s.^	1.3e−06 ± 3.4e−6^n.s.^	–
Gekkota (*n* = 63)	0.016 ± 0.012^n.s.^	−7.9e−6 ± 3.5e−5^n.s.^	−6.1e−04 ± 3.8e−3^n.s.^	−2.8e−5 ± 1.2e−4^n.s.^	−3.6e−06 ± 4.8e−6^n.s.^	–
Lacertidae (*n* = 31)	9.4e−3 ± 1.1e−2^n.s.^	2.4e−5 ± 2.6e−5^n.s.^	−1.3e−3 ± 2.4e−3^n.s.^	−1.7e−4 ± 1.4e−4^n.s.^	1.4e−6 ± 3.1e−6^n.s.^	–
Scincidae (*n* = 33)	−0.009 ± 0.015^n.s.^	−6.1e−5 ± 5.7e−5^n.s.^	−7.0e−3 ± 7.2e−3^n.s.^	−2.1e−4 ± 1.8e−4^n.s.^	**−1.6e−5 ± 8.0e−6** ^*^	–

Note

a,b: Nine and three species that were included in all lizards (*n* = 211, *n* = 155, respectively) were not included in the four major groups. * *p* < .05; ** *p* < .01; *** *p* < .001; ^n.s.^ *p* > .05. c: Nagelkerke pseudo‐*R* ^2^, but these cannot be interpreted as the percentage of variance explained by the model. d: Mixed models. Significant relationships are in boldface.

Phylogenetic signals were high in the PGLS models at the interspecific level (lambda range from 0.62 to 1, significantly different from zero at *p* < .05). For all lizards, temperature was found to be positively correlated with body mass, whereas we generally detected no significant correlations between body mass and climate variables for the four major groups (*p* > .05) (Table 2). The exception in this regard, however, was for agamids, in which temperature seasonality was observed to be negatively correlated with body mass. The GLMMs, either including or excluding species with the 10% largest range sizes, generally yielded results similar to those obtained using PGLS analysis, although only temperature was found to be positively correlated with agamid body mass (Table 2; Appendix S3).

## 4 DISCUSSION

On the basis of our analyses of a comprehensive dataset of body sizes among the species of lizard distributed in China, we arrived at the conclusion that assessments made considering all species as a single clade can be misleading (Feldman & Meiri, 2014). We found that body size trends differed depending on the level of analysis (between assemblage‐ and species‐based levels) among Chinese lizards. Whereas body size can to a certain extent be predicted by climate factors using an assemblage approach, we established that the body sizes of Chinese lizards are poorly predicted by climate using either of the assessed models based on an interspecific approach.

At the assemblage level of lizards as a whole, the geographical pattern of body size appears to be positively correlated with precipitation and NPP; however, we failed to detect consistent correlations among the four major groups. Although it is well established that body size can be negatively associated with precipitation with respect to reducing water loss (e.g., caecilian size can be promoted by aridity (Pincheira‐Donoso et al., 2019), we found no evidence in support of this pattern in either lizards as a whole or within the four family‐based groups. However, precipitation is an indicator of resources, as it can influence the abundance and distribution of vegetation, and hence the number of insects (Cesne et al., 2015), sufficient numbers of which can support large‐bodied species (Liang et al., 2018). Moreover, although we found that body size was positively correlated with NPP among all lizards, with the exception of agamids, we were unable to detect a similar pattern in the three other major groups assessed.

Conversely, whereas temperature was found to be correlated with body size in all four of the family‐based lizard groups, a similar pattern could not be detected for lizards as a whole. This could be attributable to the fact that whereas lacertid body size is negatively correlated with temperature, that of species in the remaining three groups is positively associated, which would be consistent with the “heat balance hypothesis” (i.e., size decreases in response to a reduction in temperature, as small size is conducive to rapid heat uptake; Table 2). In this regard, we speculate that the body size–temperature correlation detected for geckos is influenced by repeated species co‐occurrences, and consequently, the spatial size pattern of geckos is in need of further study. Moreover, the “heat balance hypothesis” is considered somewhat controversial (see discussion in Meiri et al., 2013; Slavenko et al., 2019), in that a larger size is also associated with a higher thermal inertia for cooling (e.g., *Psammodromus algirus*, Zamora‐Camacho et al., 2014) and hence promotes heat conservation to maintain a high body temperature in cold areas (Martin & PilarLopez, 2003; Penniket & Cree, 2015).

The “starvation resistance hypothesis” predicts that seasonal regions are conducive to large‐sized species, as larger bodies can accumulate larger amounts of nutritional reserves (Ashton & Feldman, 2003), which can ensure survival during long periods of hibernation. However, although we found that lacertid body size was positively correlated with temperature seasonality, this hypothesis may not explain the pattern of body size among Chinese lizards as a whole. In general, we found that body size in most major groups was negatively associated with either temperature or precipitation seasonality, which we speculate could be associated with their relatively short period of activity (Horváthová et al., 2013), and this in turn limits body size by influencing growth rates (e.g., *Phrynocephalus przewalskii*, see Zhao et al., 2020).

At the interspecific level, we found that the body sizes of lizards were poorly predicted by climate, which was in marked contrast to the results obtained at the assemblage level. These results did, however, still hold after omitting the 10% widest ranging species and when using all 211 species (mixed models). At the assemblage level, statistical bias (e.g., inflated type I error rates), associated with repeated species co‐occurrence, could conceivably yield meaningless statistical relationships (Hawkins et al., 2017). This would indeed appear to be true in the case of geckos, for which the observed Nagelkerke pseudo‐R^2^ values were found to be smaller than 100 random values. However, we believe that for most major groups and for lizards as a whole, the body size–climate correlations obtained are meaningful, given the larger observed Nagelkerke pseudo‐R^2^ values (which were higher than the random values). Therefore, repeated species co‐occurrences may not account for differences detected at the assemblage and species levels. Alternatively, as Feldman and Meiri (2014) have proposed, assemblage‐level analysis is essentially an ecological approach to assessing community assembly in relation to climate, whereas species‐level analysis focuses on trait evolution within a phylogenetic framework. Consequently, we assume that our use of these two different levels of analysis may be the source of the inconsistent results obtained in this study.

At the species level, temperature was found to be correlated with body size in agamids, which may have contributed to our observation of a similar correlation in lizards as a whole. In this regard, our findings are generally consistent with those reported by Guo (2016), who also established that the sizes of Chinese lizards are poorly predicted by climate at the interspecific level. Lizards can maintain a higher body temperature than the environment by behavioral adjustments, regardless of the length of time during which they are active (Meiri et al., 2013), which may thereby ensure that species do not need to modify their size to adapt to local climates (Guo, 2016).

We accordingly speculate that the size patterns in Chinese lizards are driven by other nonclimatic factors. For example, we found that the Lacertidae family of lizards is the only lineage in which body size is positively correlated with latitude (although not temperature), and thus, the potential mechanisms underlying this association cannot be explained solely in terms of Bergmann's rule. In contrast, we suggest that this pattern may be associated with the evolution of vivipary. Viviparous lineages (particularly the females of species) require larger body sizes than oviparous taxa to support the growth of embryos (Braña, 1996; Sun et al., 2012) or larger broods (but see Meiri et al., 2020) within the abdomen. Indeed, viviparous lineages tend to be distributed predominantly in cold regions ( Feldman et al., 2015), and hence, the environmental temperatures they experience are colder than those in regions inhabited by oviparous species ( Meiri et al., 2013). This may also explain why the lacertid lineage examined in the present study was the only group for which body size was positively correlated with temperature seasonality at the assemblage level. Viviparous species, such as *Zootoca vivipara*, the largest species within the family Lacertidae, are distributed at high latitudes, which may contribute to the observed geographical pattern of body size among the members of this family. Given these considerations, we believe that further studies on the geographical patterns of body size are warranted to assess a more extensive range of potential causal factors.

A further factor, activity time, can also influence correlations between climate and body size. Among Australian snakes, for example, activity time has been shown to be associated with temperature (Feldman & Meiri, 2014). This is particularly evident for nocturnal species, which may be under different selective forces compared with diurnal species, and hence, any correlation between climate and size may be weak (as they do not bask, Penniket & Cree, 2015). In the present study, geckos were the only nocturnal species examined, and we detected no obvious correlation between climate and gecko body size. We accordingly speculate that climate probably plays, at most, a minor role in shaping the size of geckos, given that they can climb in search of suitable microhabitats rather than modifying body size.

In the present study, we limited our analyses to associations between body size and climate. Other studies have, however, identified altitude as a factor associated with size (Ashton & Feldman, 2003; Jadin et al., 2019; Slavenko et al., 2021), and this may thus play a key role in shaping the geographical pattern of size among ectotherms. Accordingly, we believe the influence of altitude on the size of Chinese lizards warrants further study.

## 5 CONCLUSION

In this study, we assessed body size patterns among the most extensive range of Chinese lizards examined to date and established that spatial patterns in the body size of these lizards are driven by multiple factors, among which factors other than those relating to climate may play a prominent role in shaping body size patterns. We also suggest that further studies on altitude may contribute to gaining a more complete understanding of body size patterns in Chinese lizards.

## CONFLICT OF INTEREST

The authors declare that they have no conflict of interest.

## AUTHOR CONTRIBUTION

**Tao Liang:** Conceptualization (equal); Data curation (equal); Formal analysis (equal); Investigation (equal); Methodology (equal); Project administration (equal); Resources (lead); Software (lead); Validation (equal); Visualization (lead); Writing‐original draft (lead); Writing‐review & editing (lead). **Zi Zhang:** Data curation (supporting); Resources (supporting). **Wen‐ya Dai:** Data curation (supporting); Resources (supporting). **Lei Shi:** Conceptualization (equal); Data curation (supporting); Supervision (equal); Writing‐review & editing (supporting). **Changhu Lu:** Supervision (equal); Writing‐review & editing (supporting).

## Supporting information

Appendix S1‐S3

## Data Availability

Body size data available from Dryad (datadryad.org), https://doi.org/10.5061/dryad.j6q573ndn.
