# Supplementary material for: Spatial patterns in the size of Chinese lizards are driven by multiple factors
Source: Ecol Evol. 2021 Jun 27;11(14):9621–30. doi: 10.1002/ece3.7784 (PMC8293706; doi:10.1002/ece3.7784)
Supplement: Supplementary file 1 — Appendix S1‐S3 [file ECE3-11-9621-s001.docx]

**Appendix S1.** **Maps of the rest 46 species that were originally created as part of this study**


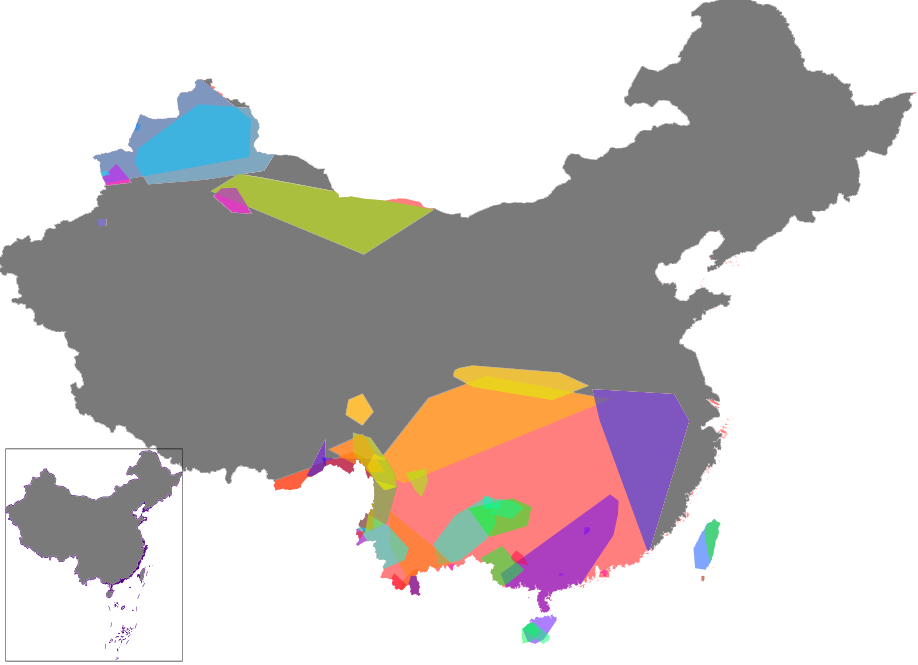


Note: 46 species names: *Acanthosaura tongbiguanensis, Cyrtodactylus cayuensis, Cyrtodactylus wayakonei, Dibamus bogadeki, Diploderma chapaense, Diploderma drukdaypo, Diploderma flaviceps, Diploderma iadinum, Diploderma laeviventre, Diploderma menghaiense, Diploderma micangshanense, Diploderma slowinskii, Diploderma swild, Diploderma vela, Eremias roborowskii, Eutropis cumingi , Eutropis multicarinata, Gekko guishanicus, Gekko kwangsiensis, Gekko liboensis, Goniurosaurus araneus, Goniurosaurus sinensis, Goniurosaurus zhoui, Hemiphyllodactylus dushanensis, Hemiphyllodactylus hongkongensis, Hemiphyllodactylus huishuiensis, Hemiphyllodactylus jinpingensis, Hemiphyllodactylus longlingensis, Lygosoma bowringii , Phrynocephalus alpherakii, Phrynocephalus grumgrzimailoi, Phrynocephalus helioscopus, Phrynocephalus melanurus, Phrynocephalus nasatus, Plestiodon leucostictus, Plestiodon liui, Pseudocalotes austeniana, Pseudocalotes kingdonwardi, Ptychozoon bannaense, Sphenomorphus tonkinensis, Takydromus albomaculosus, Takydromus yunkaiensis, Teratoscincus roborowskii, Teratoscincus scincus, Varanus irrawadicus, Varanus nebulosus*

**Appendix S2. Histograms of pseudo-R2 Nagelkerke values for spatial autoregressive models assessing associations between random body size and climate (A) and results of one sample t-test between observed nagelkerke pseudo-R^2^ and 100 random values (B)**

A

Note: A. lizards as a whole; B. Agamidae; C. Gekkota, D. Lacertidae; and E. Scincidae. The red lines indicate observed pseudo-R2 Nagelkerke values. **
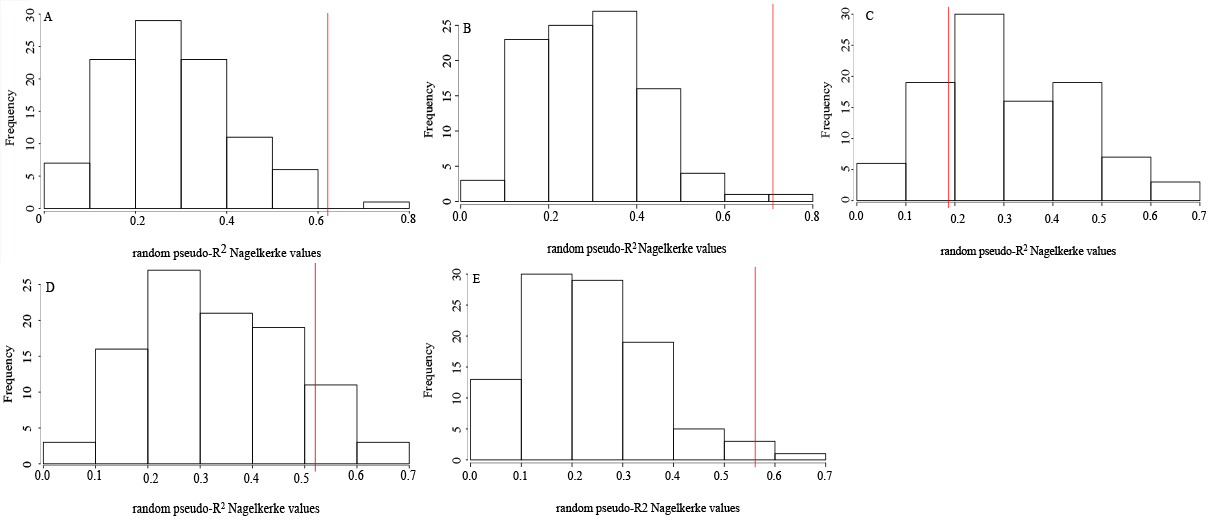
**

B

# lizards as a whole

data: nak.all.rand

t = -26.394, df = 99, p-value < 2.2e-16

alternative hypothesis: true mean is not equal to 0.6216042

95 percent confidence interval:

0.2556138 0.3067944

sample estimates:

mean of x

0.2812041

# Agamidae

t = -31.333, df = 99, p-value < 2.2e-16

alternative hypothesis: true mean is not equal to 0.7095391

95 percent confidence interval:

0.2733079 0.3252675

sample estimates:

mean of x

0.2992877

# Gekkota

t = 8.5457, df = 99, p-value = 1.603e-13

alternative hypothesis: true mean is not equal to 0.1865663

95 percent confidence interval:

0.2802346 0.3368856

sample estimates:

mean of x

0.3085601

#Lacertidae

t = -13.613, df = 99, p-value < 2.2e-16

alternative hypothesis: true mean is not equal to 0.5201696

95 percent confidence interval:

0.3012929 0.3569811

sample estimates:

mean of x

0.329137

#Scincidae

t = -26.598, df = 99, p-value < 2.2e-16

alternative hypothesis: true mean is not equal to 0.5609195

95 percent confidence interval:

0.2124457 0.2608282

sample estimates:

mean of x

0.2366369

**Appendix S3. Results of the mixed models at species-level approach after omitting the 10% largest range size.**

Results were similar to the mixed models that used all 211 species. Only temperature was correlated with body size in lizards as a whole, however, this correlation only existed in agamids but not in the rest three groups.

|  | Temperature | Temperature Seasonality | Precipitation Seasonality | Precipitation | Net primary productivity |
| --- | --- | --- | --- | --- | --- |
|  |  |  |  |  |  |
| Lizards (n=164) | 0.011±0.005^**^ | -1.6e-6±1.6e-5^n.s.^ | 1.4e-03±1.6e-3 ^n.s.^ | -3.9e-05±6.2e-5^n.s.^ | -1.4e-06±2.1e-6 ^n.s.^ |
| Agamidae (n=50) | 0.017±0.008 ^*^ | -1.2e-5±3.4e-05 ^n.s.^ | 2.1e-3±2.5e-3 ^n.s.^ | -1.9e-5±9.4e-5^n.s.^ | 1.9e-6 ±3.7e-6 ^n.s.^ |
| Gekkota (n=43) | 0.018±0.012 ^n.s.^ | 4.7e-6±4.1e-5 ^n.s.^ | -4.4e-4±4.1e-3 ^n.s.^ | -1.5e-5±1.3e-4 ^n.s.^ | -4.7e-6±5.3e-6^n.s.^ |
| Lacertidae (n=26) | 6.8e-3±1.2e-2 ^n.s.^ | 2.6e-5±2.7e-5 ^n.s.^ | 1.2e-4±2.8e-3 ^n.s.^ | -7.8e-5±1.5e-4 ^n.s.^ | 2.9e-8±3.5e-6 ^n.s.^ |
| Scincidae (n=33) | -9.3e-03±1.5e-2 ^n.s.^ | -6.9e-5±5.5e-5 ^n.s.^ | -2.7e-3±7.1e-3 ^n.s.^ | -1.2e-4±1.8e-4 ^n.s.^ | -1.3e-5±7.7e-6 ^n.s.^ |
